# Neoadjuvant toripalimab combined with chemotherapy in locally advanced HNSCC

**DOI:** 10.3389/fonc.2025.1571776

**Published:** 2025-05-19

**Authors:** Zhaoyang Wang, Sheng Yang, Dangui Yan, Ye Zhang, Xiwei Zhang, Fa Zhang, Xiaohui Zhao, Zongmin Zhang, Shaoyan Liu, Lin Gui, Changming An

**Affiliations:** ^1^ Department of Head and Neck Surgery, National Cancer Center/National Clinical Research Center for Cancer/Cancer Hospital, Chinese Academy of Medical Sciences and Peking Union Medical College, Beijing, China; ^2^ Department of Medical Oncology, National Cancer Center/National Clinical Research Center for Cancer/Cancer Hospital, Chinese Academy of Medical Sciences and Peking Union Medical College, Beijing, China; ^3^ Department of Radiation Oncology, National Cancer Center/National Clinical Research Center for Cancer/Cancer Hospital, Chinese Academy of Medical Sciences and Peking Union Medical College, Beijing, China

**Keywords:** head and neck squamous cell carcinoma (HNSCC), immunotherapy, neoadjuvant therapy, toripalimab, safety and efficacy

## Abstract

**Introduction:**

To determine the safety and efficacy of neoadjuvant toripalimab combined with chemotherapy in locally advanced head and neck squamous cell carcinoma (LA-HNSCC).

**Patients and methods:**

This single-arm investigator-initiated trial was conducted at a tertiary cancer hospital in China. The untreated LA-HNSCC patients received two cycles of neoadjuvant paclitaxel and cisplatin (TP) + toripalimab regimen (paclitaxel 175 mg/m^2^ d1, cisplatin 75 mg/m^2^ d1, and toripalimab 240 mg d1, every 3 weeks). Surgery or chemoradiotherapy (CRT) was determined via the patient–doctor consensus. The primary endpoints were objective response rate (ORR) and safety.

**Results:**

The study enrolled a total of 23 patients. All the patients completed the entire treatment course with an ORR of 78.3% (18/23). Any grades of treatment-related adverse events (TRAEs) were reported in 12 patients (52.2%), and three patients experienced grade 3–4– TRAEs. No delay for curative treatment was observed. Twelve patients underwent radical surgery, and six patients developed pathological complete response (pCR), with a pCR rate of 50%. With a median follow-up of 15 months, 12 surgery patients maintained event-free survival; however, three out of 11 who received CRT suffered from local recurrence or metastases.

**Conclusions:**

Neoadjuvant TP + toripalimab for LA-HNSCC showed high ORR and pCR rates with a good safety profile.

**Clinical trial registration:**

https://www.chictr.org.cn/showproj.html?proj=231832, identifier ChiCTR2400091148.

## 1 Introduction

Immunotherapy in head and neck squamous cell carcinoma (HNSCC) is a rapidly evolving field in the last decades. Based on several large clinical trials such as CheckMate-141 (1), KEYNOTE-012 (2), and KEYNOTE-048 (3), immune checkpoint inhibitors (ICIs) against programmed death-1 (PD-1) have become the first-line treatment for recurrent/metastatic head and neck squamous cell carcinoma (r/m HNSCC).

The successful application of immunotherapy in r/m HNSCC has stimulated interest in its neoadjuvant treatment due to its potential value in reducing local recurrence and distant metastases and improving organ preservation. Several ongoing neoadjuvant clinical trials have shown promising results, including mono- or dual immunotherapy and chemoimmunotherapy (4–8).

Toripalimab is a recombinant, humanized PD-1 monoclonal antibody with promising clinical activity and tolerable safety across several cancers. It has been approved for recurrent/metastatic nasopharyngeal cancer in China and the USA. Currently, there is a lack of studies regarding the application of toripalimab in the neoadjuvant treatment of HNSCC. Here, we introduced the results of an investigator-initiated trial evaluating neoadjuvant toripalimab combined with chemotherapy in patients with locally advanced HNSCC (LA-HNSCC).

## 2 Materials and methods

### 2.1 Patient eligibility

This single-arm investigator-initiated trial was conducted at the National Cancer Center/Cancer Hospital, Chinese Academy of Medical Sciences, and Peking Union Medical College. The trial was approved by the ethics committee of the National Cancer Center (NCC2020C-471). The inclusion criteria were as follows: aged 18 to 75 years, pathologically confirmed HNSCC (oral cavity, oropharynx, larynx, hypopharynx, and cervical esophagus), resectable locally advanced stage (T3-4Nx and TxN1-3), no prior anti-cancer therapy before enrollment, p16 positive or negative, expected survival over 6 months, no contraindication for immunotherapy or chemotherapy, an Eastern Cooperative Oncology Group (ECOG) performance status score of 0 to 2, and normal organ function. The primary exclusion criteria included unresectable disease, distant metastasis detected, active viral hepatitis, and immunodeficiency status. Written informed consent was obtained from all patients before clinical treatment according to institutional guidelines.

### 2.2 Trial design

All patients received paclitaxel and cisplatin (TP) + toripalimab neoadjuvant regimen for two cycles (paclitaxel 175 mg/m^2^ d1, cisplatin 75 mg/m^2^ d1, and toripalimab 240 mg d1, every 3 weeks). Toripalimab infusion should precede chemotherapy with a minimum interval of 1 hour. In subsequent treatment cycles, a minimum 30-minute interval should be maintained between administrations. Preventive antiemetics, liver protection, hydration, and other symptomatic treatments were given. After neoadjuvant therapy, the choice of radical surgery or chemoradiotherapy (CRT) was determined by the patient–doctor consensus. The curative treatment was performed 1–3 weeks after the completion of neoadjuvant treatment. The surgical plan was designed according to the extent of the tumor before neoadjuvant treatment, and the corresponding surgical methods were selected according to the different parts of the tumor. Neck dissection was performed simultaneously. The surgical team was composed of senior doctors in the study group, and the differences in surgical quality were minimized through standardized preoperative planning, intraoperative on-site communication, and uniform recovery pathways to achieve the consistency of surgical standards.

The multidisciplinary team (MDT) played a pivotal role in the clinical trial, engaging in all stages of the trial. This comprehensive involvement encompassed critical aspects such as patient diagnosis, formulation of neoadjuvant treatment protocols, determination of the optimal therapeutic approach through surgery or radiotherapy, and subsequent postoperative care management. The MDT’s continuous engagement ensures a seamless integration of multifaceted expertise, thereby enhancing the precision and personalization of clinical care throughout the trial.

### 2.3 Endpoints and efficacy and safety assessment

The primary endpoint was objective response rate (ORR) and safety. The secondary endpoints were as follows: 2-year and 5-year overall survival (OS), 2-year and 5-year progression-free survival (PFS) rates, pathological response, treatment completion rate, and treatment-related adverse events (TRAEs). In the current study, the duration was not sufficient to warrant the inclusion of OS or PFS analyses in the manuscript.

The clinical responses were evaluated according to Response Evaluation Criteria in Solid Tumors (RECIST) guidelines version 1.1 (9). Computed tomography (CT) and magnetic resonance imaging (MRI) were used for measuring the lesions’ sum of the longest diameters (SLD).

The adverse events were recorded in all patients based on the National Cancer Institute Common Terminology Criteria for Adverse Events, version 5.0 (CTCAE 5.0). The surgical delay was defined as the interval between the date of surgery and the last neoadjuvant medication being more than 6 weeks.

## 3 Results

### 3.1 Demographic characteristics

**Figure 1:**
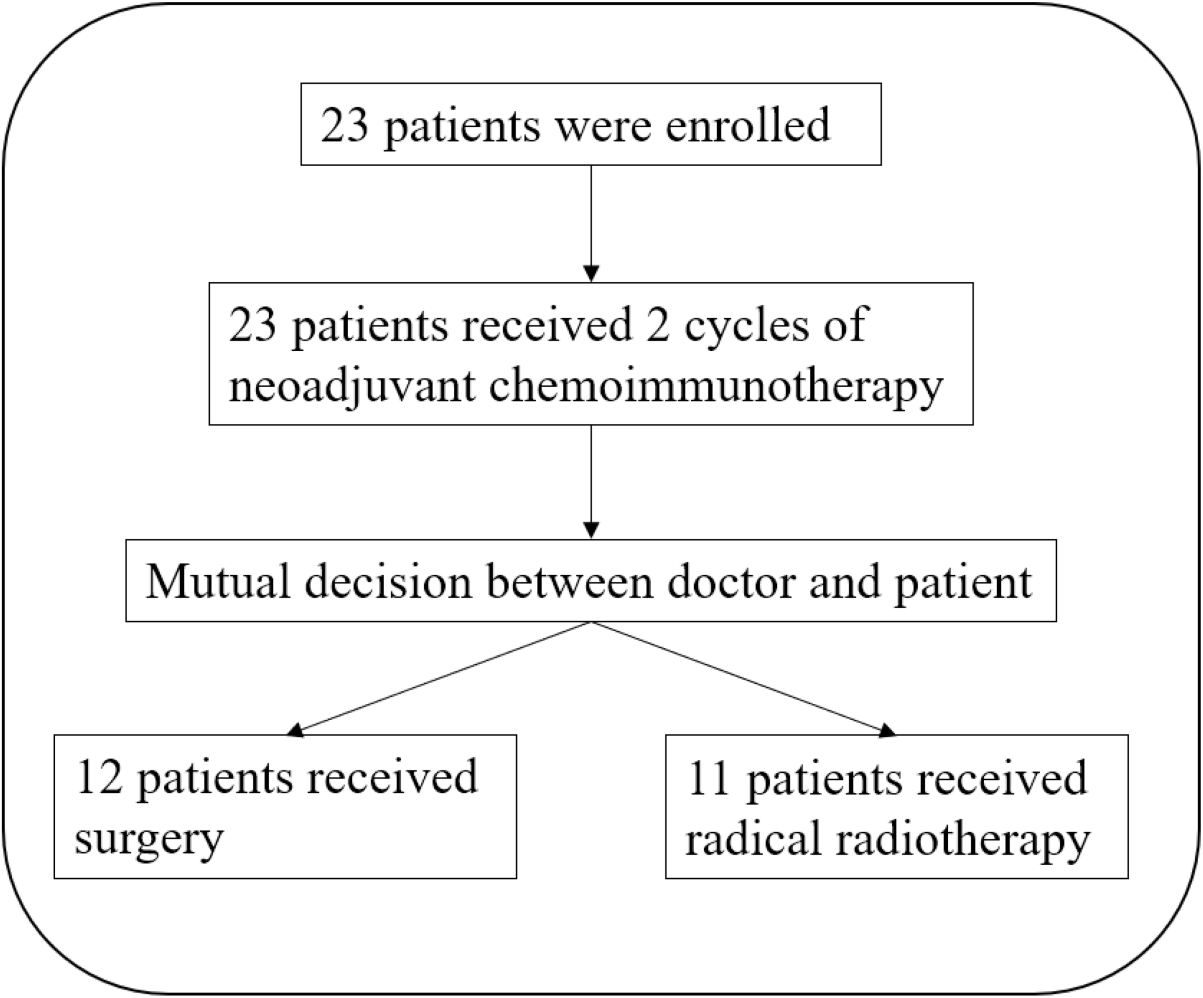
Patient flowchart.

From February 2021 to May 2023, a total of 23 patients received the TP + toripalimab neoadjuvant regimen, and 12 patients received radical surgery (**Figure 1**). Demographic and clinical characteristics are shown in **Table 1**. The median age was 58 years, and 21 were male. Primary tumors were located in the following: one at the oral cavity, 10 at the oropharynx, six at the hypopharynx, four at the larynx, and two at the cervical esophagus. Eight out of 10 oropharynx cancer patients were p16 positive by immunohistochemistry.

**Table 1:** Demographic characteristics.

Variable	Overall (N = 23)
Gender n (%)	
Male	22 (95.7%)
Female	1 (4.3%)
Age (years)	
Median [min, max]	58.0 [34.0, 75.0]
Smoker	
<10 pack-years	8 (34.8%)
≥10 pack-years	15 (65.2%)
Alcohol consumption	
Light/moderate drinking	12 (52.2%)
Heavy drinking	11 (47.8%)
Primary tumor sites	
Oral cavity	1 (4.3%)
P16+ oropharynx	8 (34.8%)
P16− oropharynx	2 (8.7%)
Larynx	4 (17.4%)
Hypopharynx	6 (26.1%)
Cervical esophagus	2 (8.7%)
Pre-treatment T stage	
T1	4 (17.4%)
T2	7 (30.4%)
T3	4 (17.4%)
T4	8 (34.8%)
Pre-treatment N stage	
N0	2 (8.7%)
N1	5 (21.7%)
N2	16 (69.6%)
American Joint Committee on Cancer (AJCC) TNM stage	
I	4 (17.4%)
II	2 (8.7%)
III	3 (13.0%)
IV	14 (60.9%)
CPS	
≥20	10 (43.5%)
[10, 20)	5 (21.7%)
[5, 10)	4 (17.4%)
[1, 5)	4 (17.4%)
p16	
Negative	14 (60.9%)
Positive	9 (39.1%)
Treatment after neoadjuvant therapy	
Surgery	12 (52.2%)
Radiotherapy	11 (47.8%)

CPS, combined positive score.

A total of 23 baseline samples were evaluated for the treatment parameters. Combined positive scores (CPSs) ranged from 1 to 100. Of the patients, 43.5% (n = 10) had CPS ≥ 20, and 56.5% (n = 13) had CPS between 1 and 19.

### 3.2 Safety analysis

All 23 patients completed two cycles of TP + toripalimab neoadjuvant chemoimmunotherapy. The TRAEs are summarized in **Table 2**. Any grades of TRAEs were reported in 12 patients (52.2%), and four patients experienced ≥2 concurrent TRAEs. The most common TRAEs were leukopenia, anemia, and inappetence, in order. Three patients (13.0%) experienced grade 3–4– TRAEs, and all of them were leukopenia. There was no delay for curative treatment due to TRAEs after appropriate treatment.

**Table 2:** Neoadjuvant toripalimab combined with TP TRAEs.

Events	Grade 1–2	Grade 3	Grade 4
All patients with TRAEs	9	1	2
Leukopenia	3	1	2
Anemia	6	0	0
Inappetence	4	0	0
Nausea/vomiting	3	0	0
Myocardial infarction	1	0	0
Thyroid dysfunction	1	0	0

TP, paclitaxel and cisplatin; TRAEs, treatment-related adverse events.

### 3.3 Efficacy analysis

**Figure 2:**
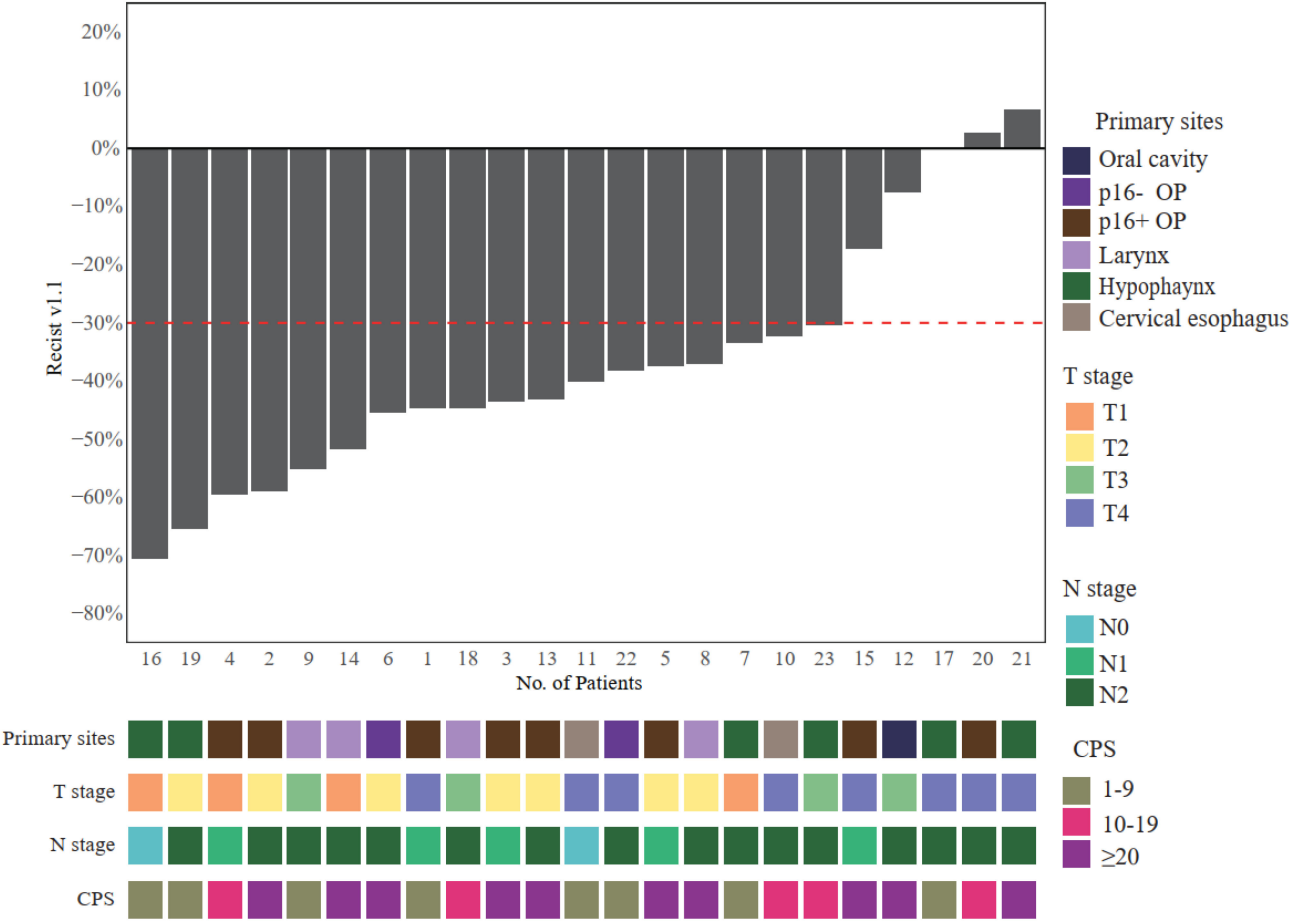
Radiological responses after neoadjuvant toripalimab + TP. OP, oropharynx; TP, paclitaxel and cisplatin.

After the neoadjuvant TP + toripalimab, clinical responses were evaluated according to RECIST 1.1 (**Figure 2**). Of 23 patients, 18 achieved partial response (PR) after neoadjuvant therapy, with an ORR of 78.3% (95% CI: 61.4%–95.1%). The other five patients had stable disease (SD). No progressive disease was observed. All the 12 patients who underwent radical surgery had R0 resection. Eight of 12 achieved major pathological response (MPR; 66.7%), including six with pathological complete response (pCR) (50%). Particularly, all five p16-positive oropharyngeal cancer patients reached pCR.

**Figure 3:**
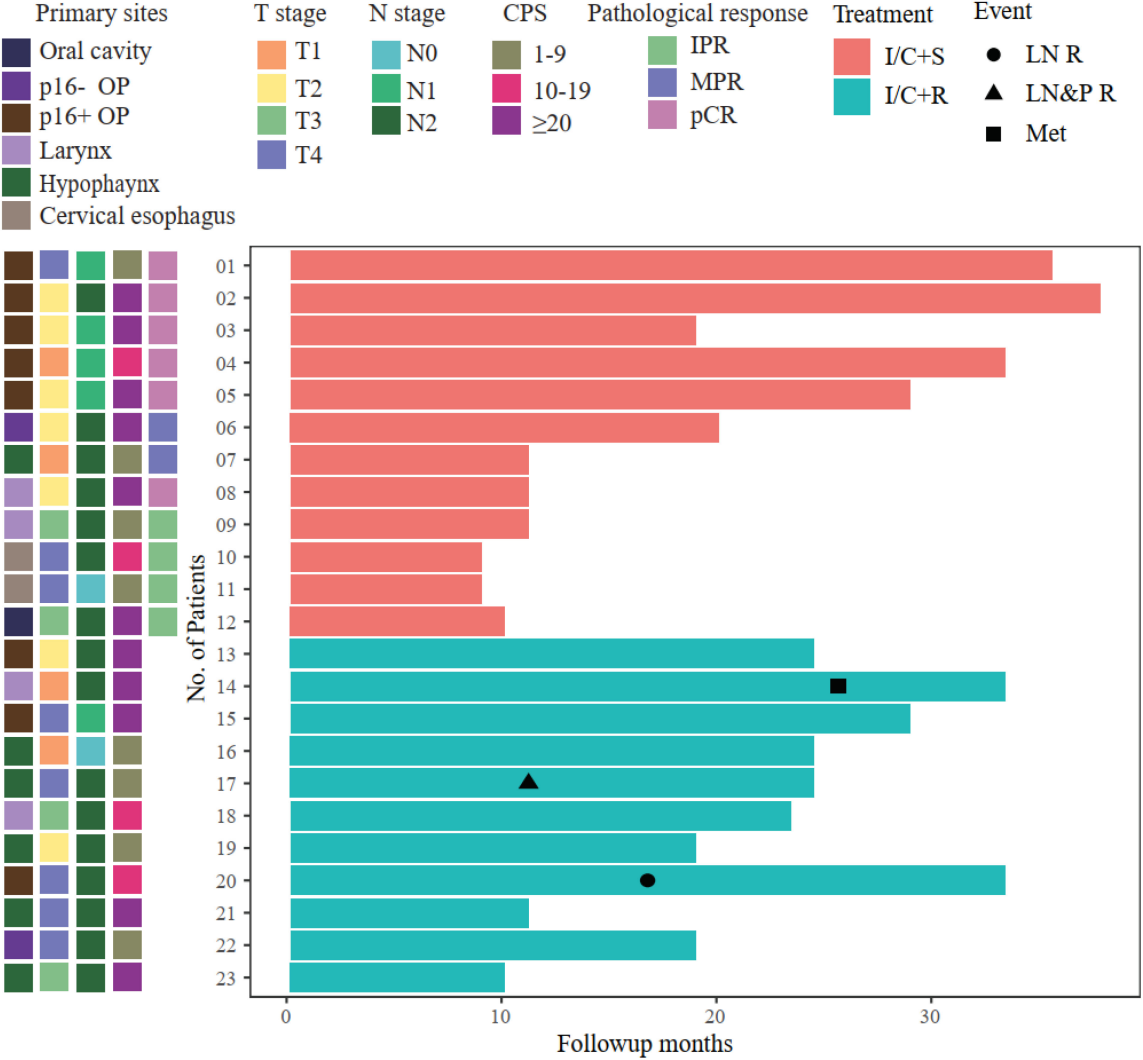
Swimmer plot illustrating patient follow-up. All the surgery patients maintained event-free survival; however, 3/11 radiotherapy patients had local recurrence or metastasis. OP, oropharynx; IPR, incomplete pathological response; MPR, major pathological response; pCR, pathological complete response; I/C+S, immunochemotherapy followed by surgery; I/C+R, immunochemotherapy followed by radiotherapy; LN R, lymph node recurrence; LN&P R, lymph node and primary site recurrence; Met, metastasis.

Six patients with pCR did not receive postoperative radiotherapy based on multidisciplinary team discussion. Instead, they were treated with toripalimab 240 mg every month for 6 to 12 months (**Figure 3**). The other six patients who did not achieve pCR received postoperative radiotherapy as routine therapy.

Potential factors associated with pCR were analyzed (**Table 3**). Positive p16 status was a significant pCR predictive factor (*p* = 0.015). CPS ≥ 20 had a higher pCR rate (66.7% vs. 33.3%), but the difference was not statistically significant (*p* = 0.284). No factors other than p16 status showed a significant association with pCR.

**Table 3:** pCR correlation analysis.

Variable	pCR	Non-pCR	*p*-Value
Age (mean ± SD, years)	51.7 ± 10.8	63.5 ± 7.3	0.051
Gender			
Male	6 (100.0%)	0 (0%)	0.296
Female	5 (83.3%)	1 (16.7%)	
P16 status			
Positive	5 (83.3%)	1 (16.7%)	0.015*
Negative	0 (0%)	6 (100.0%)	
Smoker			
≥10 pack-years	2 (28.6%)	5 (71.4%)	0.242
<10 pack-years	4 (80.0%)	1 (20%)	
Alcohol consumption			
Heavy drinking	3 (42.9%)	4 (57.1%)	0.558
Light/moderate drinking	3 (60.0%)	2 (40.0%)	
T stage			
T1–2	5 (71.4%)	2 (28.6%)	0.242
T3–4	1 (20.0%)	4 (80.0%)	
N stage			
N0–1	4 (80.0%)	1 (20.0%)	0.242
N2	2 (28.6%)	5 (71.4%)	
Primary sites			
Oral cavity	0 (0%)	1 (100.0%)	0.155
Oropharynx	5 (83.3%)	1 (16.7%)	
Larynx	1 (50.0%)	1 (50.0%)	
Hypopharynx	0 (0%)	1 (100.0%)	
Cervical esophagus	0 (0%)	2 (100.0%)	
CPS ≥ 20			
Yes	4 (66.7%)	2 (33.3%)	0.284
No	2 (33.3%)	4 (66.7%)	
CPS ≥ 10			
Yes	5 (62.5%)	3 (37.5%)	0.273
No	1 (25.0%)	3 (75.0%)	
SLD change	46.8% ± 10.1%	35.6% ± 16.1%	0.178

pCR, pathological complete response; CPS, combined positive score; SLD, sum of the longest diameters.

*, *p*<0.05.

The median follow-up time was 15 months (ranging from 5 to 31 months). Twelve patients who underwent surgery maintained event-free survival. However, among 11 patients who accepted definite radiotherapy, two patients had cervical lymph node recurrence, and one patient suffered brain metastases (**Figure 3**). Patient 17 had hypopharyngeal SCC (T4aN2b). After neoadjuvant therapy, the radiological response was SD; then, he received concurrent platinum-based chemoradiotherapy. However, he had recurrence at primary sites and regional lymph nodes after 6 months from the end of the radiotherapy. Then, he received chemotherapy based on TP plus cetuximab, and the tumor load was stable by the cutoff date of follow-up. Patient 20 had p16-positive oropharyngeal SCC, and the radiological assessment after neoadjuvant therapy was SD. Then, he received concurrent platinum-based chemoradiotherapy, and he was found to have recurrence at regional lymph nodes at 9 months after the adjuvant therapy. He received the salvage neck dissection without other systemic medication and maintained disease-free survival by the cutoff date. Patient 14 had supraglottic laryngeal SCC (T1N2b), and the radiological response was PR after neoadjuvant therapy. He refused surgery and received concurrent chemoradiotherapy. The patient was found to have brain metastases due to central nervous system symptoms at a local hospital. The patient was treated with anlotinib and toripalimab and survived by the cutoff date.

## 4 Discussion

Neoadjuvant chemotherapy is a standard treatment for patients with resectable locally advanced head and neck squamous cell carcinoma that aims to reduce the tumor burden and improve surgical outcomes. However, neoadjuvant chemotherapy alone has limited efficacy and may induce resistance to subsequent therapies. Neoadjuvant chemoimmunotherapy is a novel strategy that combines chemotherapy and immunotherapy before surgery to enhance the anti-tumor immune response and overcome the resistance mechanisms. In general, the neoadjuvant combination of toripalimab plus TP was shown to be safe and feasible in HNSCC. After two cycles of neoadjuvant chemoimmunotherapy, the most common TRAEs were anemia (6/23, 26.1%) and leukopenia (6/23, 26.1%), and the only grade 3–4– TRAE in this study was leukopenia (3/23, 13.0%). There was no delay in curative therapy.

The data on pathological complete response rates varied across the reported studies with different neoadjuvant regimens. Ferris (5) reported 52 resectable HNSCC patients who received neoadjuvant nivolumab alone. In the human papillomavirus (HPV)-positive group (n = 17), one case (5.9%) achieved MPR, and three cases (17.6%) achieved partial pathological response. In the HPV-negative group (n = 17), one case (5.9%) achieved pCR. Zinner (10) investigated the efficacy of nivolumab combined with paclitaxel and carboplatin in 27 HNSCC patients. Of the patients, 42% achieved pCR, and the incidence of TRAEs above grade 3 was 37%. Huang (8) reported 23 patients who received neoadjuvant toripalimab with gemcitabine and cisplatin. The ORR reached 45%, and the pCR and MPR were 26.7% and 27.8%, respectively. Compared to neoadjuvant immunotherapy alone, neoadjuvant immunochemotherapy resulted in preferable pathological response and manageable adverse effects. As far as we know, the pCR and MPR of neoadjuvant immunotherapy alone were 0%–16.7% and 2.9%–31.0%, respectively (5, 11–13). The pCR and MPR of immunotherapy combined with chemotherapy were 16.7%–56.0% and 27.8%–74.1%, respectively (7, 8, 14). Our study showed 50% pCR rates and 66.7% MPR rates, and it demonstrated to have relatively good efficacy compared to past studies.

Deciphering factors predicting/affecting the efficacy of neoadjuvant immunotherapy was of great importance to patient stratification and precise treatment. Positive p16 status was a significant pCR predictive factor in our study. P16-positive HNSCC may have a lower mutational burden and higher immunogenicity than p16-negative HNSCC (15, 16). Previous reports (5) indicated that p16-positive HNSCC had a better response to immunotherapy. CPS was the most widely used biomarker for predicting the response of anti-PD-1 immunotherapy (1–3, 17). Although the CPS ≥ 20 subgroup exhibited a numerically higher pCR rate compared to CPS < 20 (66.7% vs. 33.3%), this difference did not reach statistical significance (*p* = 0.284). Previous studies (18, 19) have investigated potential biomarkers such as tumor mutational burden and CD8+TiL, but further efforts are needed to build a comprehensive predictive stratification.

**Figure 4:**
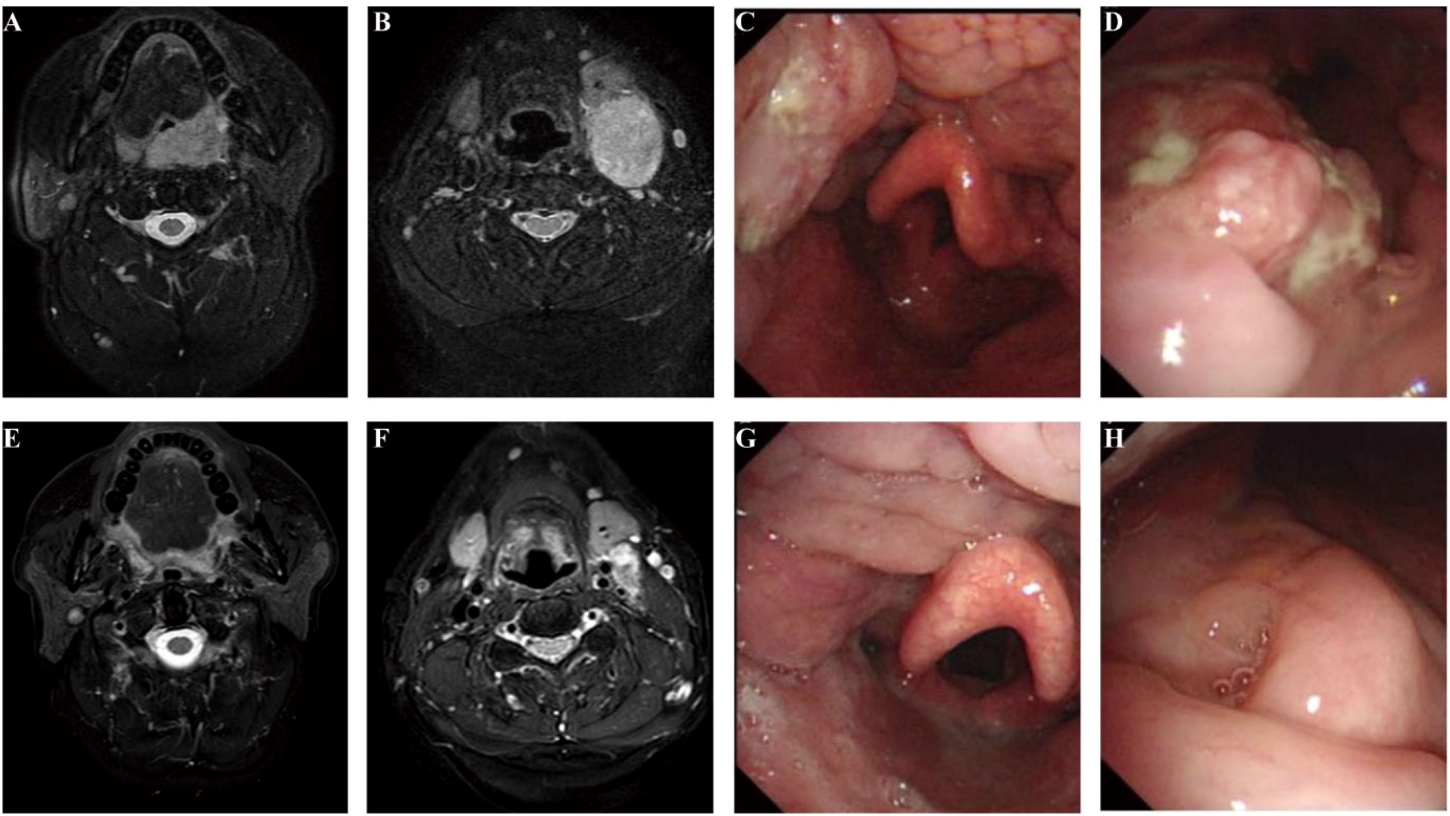
Accurate assessment of the response to neoadjuvant therapy remained challenging. Patient 5 baseline MRI **(A, B)**, endoscopy **(C, D)**, and examination after neoadjuvant treatment **(E–H)**. The SLD change was only 37.3%, which was evaluated as PR according to RECIST v1.1, but the patient achieved pCR after the surgery. SLD, sum of the longest diameters; PR, partial response; RECIST, Response Evaluation Criteria in Solid Tumors; pCR, pathological complete response.

An accurate evaluation of treatment response after neoadjuvant therapy is important for clinical decision-making. The discordance between radiological response and pathological response was noted in our study. In six pCR patients, the mean radiological response was 46.8% (ranging from 37.3% to 59.5%). We believe that two factors may contribute to the discordance. First, like most solid tumors, the residual lesions on imaging could be ascribed to the infiltration of immune cells, tumor necrosis, hemorrhage, and edema (20). Second, but particularly, it may be caused by the peculiar complex anatomy and oncogenesis of head and neck tumors. HNSCC usually originates from the epithelial layer of the upper aerodigestive tract, which contains folds and a larger mucosal surface area. After neoadjuvant therapy, the tumor mass could not shrink in a centralized pattern on the imaging, but sometimes, a flattened multi-focal tumor remained distributed as a spotty pattern. How to accurately evaluate tumor regression after neoadjuvant therapy is of great importance to the clinical decision in HNSCC. For example, patient 5 had p16-positive T2N1 oropharyngeal cancer (**Figure 4**). The SLD change was only 37.3% after neoadjuvant therapy, but the patient achieved pCR after the surgery. Circulating tumor DNA (ctDNA) may be a promising approach. ctDNA has been shown to be a good reflection of tumor burden in a variety of tumor types (21, 22), and the change in abundance through neoadjuvant therapy could be a potential marker for evaluating tumor regression. Our group has already carried out related work, and we were expecting the subsequent results to provide more precise treatment options for HNSCC patients.

After neoadjuvant therapy, the choice between surgical and non-surgical treatments remained inconclusive in HNSCC. By the date of cutoff, there were no recurrences in the surgery subgroup, whereas three patients suffered from local recurrence or metastases in the CRT group. To robustly assess the comparative efficacy of surgery versus radiotherapy after neoadjuvant chemoimmunotherapy, future studies should incorporate extended follow-up durations and expanded patient populations. To the best of our knowledge, this was the first report concerning the comparison between surgery and radiotherapy after neoadjuvant immunotherapy. However, further patient enrollment and follow-up were needed to build a solid conclusion.

In conclusion, neoadjuvant toripalimab combined with chemotherapy for locally advanced HNSCC showed high ORR and pCR rates, with a good safety profile. In early follow-up, all the surgery patients maintained event-free survival. Our study encouraged further large-scale clinical trials on neoadjuvant treatment strategies in HNSCC.

## Data Availability

The raw data supporting the conclusions of this article will be made available by the authors, without undue reservation.
